# Sex Differences in Maturation and Function of Neonatal Porcine Islets Upon Transplantation in Mice

**DOI:** 10.1111/xen.70039

**Published:** 2025-04-17

**Authors:** Nerea Cuesta‐Gomez, Chelsea Castro, Mandy Rosko, Karen Seeberger, Gregory S. Korbutt

**Affiliations:** ^1^ Department of Surgery University of Alberta Edmonton Alberta Canada; ^2^ Alberta Diabetes Institute University of Alberta Edmonton Alberta Canada

**Keywords:** biological variables, diabetes, islet transplantation, neonatal porcine islets, sex differences, xenotransplantation

## Abstract

**Background:**

Neonatal porcine islets (NPIs) can mature into a mixed population of endocrine cells that can restore glucose control in mice, pigs, and non‐human primates, representing a potential alternative islet source for clinical beta cell replacement therapy. However, it remains unclear how conditions in the recipient influence the maturation and function of these cells. Here, we investigated the impact of host sex on NPIs implanted under the kidney capsule of male and female B6.129S7‐Rag1^tm1Mom^ (B6/Rag^−/−^) mice.

**Methods:**

Diabetic mice were transplanted with 3000 NPIs under the kidney capsule. All mice were monitored for reversal of hyperglycemia and glucose clearance at 8‐ and 20‐weeks post‐transplant. Grafts were assessed for cell composition and insulin content.

**Results:**

Female mice demonstrated improved glucose clearance at 8‐ and 20‐weeks post‐transplant compared to their male counterparts. Improved glucose clearance correlated with accelerated diabetes reversal in females (8 weeks vs. 12 weeks in males) and increased rates of euglycemic achievement (17/18 in females vs. 14/19 in males). However, grafts collected from male mice exhibited an increased percentage of insulin‐positive cells as well as increased insulin content.

**Conclusion:**

The sex of the host influences the outcomes of NPI transplantation, showcasing the relevance of understanding the role of sex as a biological variable in islet transplantation.

## 1 Introduction

Islet transplantation (ITx) has established that cellular therapies can improve glycemic control in a subset of patients with type 1 diabetes, but it is limited by the shortage of adequate donor islets and need for chronic immunosuppression [1, 2]. Alternatively, other sources of ß‐cells have been highly sought after, including stem cell‐derived ß‐cells and xenogeneic islet alternatives. Through the recapitulation of pancreatic development, several groups have been able to generate islet‐like cells from pluripotent stem cells, and clinical trials have demonstrated C‐peptide secretion following transplant of embryonic stem cell‐derived pancreatic progenitors that mature into islets in vivo, as well as fully differentiated islets [3, 4, 5]. Despite the success of these trials, the clinical translation of stem cell‐derived islets remains limited by the need for cost‐efficient cell manufacturing process and, more importantly, concerns over the safety of the cell product due to the teratogenic potential of proliferative off‐target cell populations during differentiation [6, 7, 8, 9]. For this reason, xenotransplantation offers a promising alternative as a readily available source of islets for ITx.

The pig represents the most probable source of islets for xenotransplantation due to its unlimited availability, high breeding potential, large number of offspring, and overall close anatomical and physiological similarity to humans [10]. Moreover, porcine insulin differs by only a single amino acid from that of human insulin, and it was administered to treat diabetes for nearly a century before the introduction of recombinant human insulin, making porcine islets an ideal source for ITx [11]. However, xenozoonoses and xenoantigens were considered major concerns for clinical translation until recently, when two gene‐edited pig heart transplants were successfully carried out in living patients [12, 13]. In addition, genetically modified pig kidneys have been implanted into two brain‐dead human recipients [14]. Although adult porcine pancreases provide a source of fully mature islets, the cost of maintaining a herd of adult pigs and the poor reproducibility of isolating adult porcine islets make neonatal porcine islets (NPIs) a more reasonable source of xenogeneic ß‐cell grafts [15].

Animal studies indicate that NPIs can mature into a mixed population of endocrine cells, including glucose‐responsive beta cells several months after implantation that can reverse diabetes in vivo. However, it remains unclear how the impact of host sex may influence the maturation and ultimately the function of these cells. Studies using stem cell‐derived islets have shown accelerated islet maturation in female recipients compared to males. Investigating the relevance of host sex in ITx is vital for addressing sex‐specific disparities in health outcomes. Herein, we aim to elucidate the impact of host sex on NPI maturation and diabetes reversal.

## 2 Materials and Methods

### 2.1 Neonatal Porcine Islet Isolation and Culture

Animal use was in accordance with the guidelines approved by the Canadian Council on Animal Care. Porcine donor pancreases were surgically removed from 3 to 5‐day‐old Duroc neonatal piglets from the University of Alberta's Swine Research and Technology Center (1.5–2.0 kg body weight). NPIs were isolated and initially cultured overnight in Ham F10 media (Sigma‐Aldrich) with 666 µL/L protease inhibitor P8340 (Sigma‐Aldrich) and 5 µg/mL FMK001 caspase inhibitor (R&D systems), as described previously [16]. After 24 h, NPIs were resuspended in Ham's F10 media with 10 µM ROCK inhibitor Y27632 (Sigma‐Aldrich) and incubated for 2 days. On Day 3 of culture post‐isolation, NPIs were washed in Hank's Balanced Salt Solution (Sigma‐Aldrich) and cultured in supplemented Dulbecco's modified Eagle medium nutrient mixture F‐12 (DMEM/F12; Thermo Fisher) containing 1% pig serum and 1 × Insulin Transferrin Selenium (Thermo Fisher), supplemented with 10 mM nicotinamide and 10 nM exendin‐4 (Sigma‐Aldrich). Half media changes were conducted every 2 days to minimize NPI loss prior to transplant on Day 7. Six NPI preparations were used, with a total of 20 piglets, balanced for sex, for NPI isolations. In all cases, an NPI preparation included all NPIs isolated from piglets on the same day, regardless of the sex of the donor, to avoid donor‐associated sex‐related differences.

### 2.2 Transplantation and Metabolic Follow‐Up

Male and female B6.129S7‐Rag1^1Mom^ (B6/Rag^−/−^) mice (Jackson Laboratory) were maintained in a pathogen‐free, climatized environment, with free access to pelleted food and water containing Novo‐Trimel. Animal use was in accordance with the Canadian Council on Animal Care and approved by the institutional animal ethics committee at the University of Alberta, Edmonton AB, Canada (AUP0000278). Equal numbers of male and female mice were utilized.

Prior to transplantation, recipient mice were rendered diabetic by chemical induction, via intraperitoneal streptozotocin (Sigma‐Aldrich), dissolved in acetate buffer (pH 4.5), at 185 mg/kg. Animals were considered diabetic following a non‐fasting blood glucose measurement of ≥18.0 mmol/L on two consecutive days. Only animals meeting this inclusion criterion were selected for NPI recipients. NPIs were counted and transplanted under the left kidney capsule at a dose of 3000 islet equivalents per diabetic recipient. Mice were anesthetized with 3% isoflurane, and buprenorphine (0.1 mg/kg subcutaneous) was administered for post‐operative analgesia. Mice were assessed daily for humane endpoints. Non‐fasting blood glucose measurements were made weekly following transplant. Graft function and reversal of diabetes was defined as two consecutive readings <11.1 mmol/L which was maintained until study completion. Serum samples were collected for the presence of graft‐specific circulating porcine insulin at 8 and 20 weeks post‐transplant. Basal (0 min, fasting) and stimulated (60 min following intraperitoneal D‐glucose administration, 3 mg/g) porcine insulin levels were measured in recipient mouse serum using ALPCO ultra‐sensitive ELISA (ALPCO). Intraperitoneal glucose tolerance tests (IPGTT) were also conducted at 8 and 20 weeks posttransplant. After a 12‐h fast, D‐glucose (3 mg/g) was administered intra‐peritoneally and subsequently blood samples were obtained from the tail vein at 0, 15, 30, 60, 90, and 120 min. At 20 weeks post‐transplantation, all mice underwent a survival nephrectomy of the graft‐bearing kidney to confirm graft‐dependent euglycemia. Nephrectomized animals were subsequently monitored for 1 week to confirm return to a hyperglycemic state. Procured grafts underwent morphological analysis or assessment of cellular insulin content [16].

### 2.3 Characterization of the Harvested Grafts

Tissue cross‐sections (5 µm) were deparaffinized, rehydrated, and blocked with 20% normal goat serum (Sigma) for 1 h at room temperature and incubated with primary antibodies overnight at 4°C. Secondary antibodies were incubated for 1 h at room temperature in the dark. Antibodies and concentrations used are listed in Table 1. Samples were then washed in PBS, followed by nuclei counter‐staining with DAPI in an anti‐fade mounting medium (ProLongGold, Thermo Fisher Scientific). Slides were visualized using the Zeiss Observer Z1 inverted fluorescence microscope and images were processed using Zeiss software and analyzed using QuPath. The grafts were also measured for total cellular insulin content based on the previously described methodology [16]. Extracted kidneys were homogenized and sonicated at 4°C in 10 mL of 2 mmol/L acetic acid (containing 0.25% BSA). After 2 h at 4°C, tissue homogenates were resonicated and centrifuged (10 000 *g*, 25 min), and supernatants were collected. Pellets were then further extracted by sonication in an additional 5 mL of acetic acid. The second supernatant was collected after centrifugation, combined with the first supernatant, total volume was measured, and samples were assayed for insulin content (MesoScale Mouse/Rat Insulin Kit).

**TABLE 1:** Antibodies and concentrations used for immunohistochemistry.

Epitope	Origin animal	Dilution	Supplier
Insulin	Guinea pig	1:5	Dako (A0564)
Glucagon	Mouse	1:5000	Sigma (G2654)
Anti‐guinea pig	Goat	1:200	Invitrogen (A11073)
Anti‐mouse	Goat	1:200	Invitrogen (A11032)

### 2.4 Statistical Analysis

Normality testing was performed using the D'Agostino‐Pearson normality test, which determined the need for parametric testing. Between‐group comparisons were carried out using the unpaired *t*‐test with Welch's correction. Kaplan‐Meier survival curves were compared via log‐rank statistical testing (Mantel‐Cox). Values are presented as means with standard deviation. The alpha value was set at 0.05. All statistical analysis was completed using GraphPad Prism version 9.3.1 for Mac, GraphPad Software, www.graphpad.com.

## 3 Results

### 3.1 Transplantation of Neonatal Porcine Islets Results in Accelerated Diabetes Reversal in Females Compared to Males

To assess the impact of host sex in NPI function, 19 male and 18 female diabetic B6.129S7‐Rag1^1Mom^ (B6/Rag^−/−^) mice were used for NPI recipients. NPIs were counted and transplanted under the left kidney capsule at a dose of 3000 islet equivalents per diabetic recipient, and mice were monitored for 21 weeks to assess NPI function and diabetes reversal. Blood glucose was measured weekly, and glucose challenges were performed at 8‐ and 20‐weeks post‐transplant. Representative image and size distribution of NPIs can be found in Figure S1A,B.

**FIGURE 1:**
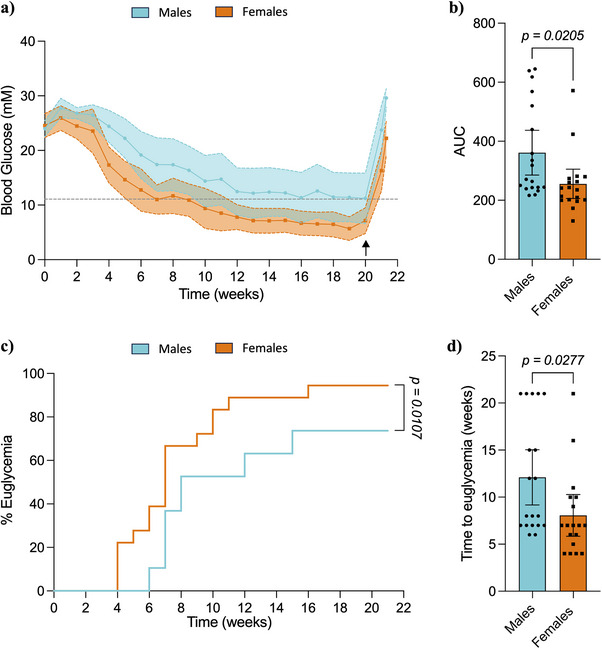
Metabolic follow‐up of diabetic male and female B6.129S7‐Rag1^tm1Mom^ (B6/Rag^−/−^) mice transplanted with neonatal porcine islets under the kidney capsule. (a) Weekly blood glucose measurements throughout the follow‐up period. Black arrow indicates graft retrieved, at which point mice reverted back to a hyperglycemic state. (b) Significance of weekly blood glucose values determined by area under the curve analysis. (c) Percentage of euglycemic animals throughout the follow‐up period. (d) Representation of time (weeks) to achieve euglycemia. Statistical difference amongst groups was conducted using the unpaired *t*‐test with Welch's correction. Kaplan‐Meier survival curves were compared via log‐rank statistical testing (Mantel‐Cox). Values are presented as means with standard deviation.

Non‐fasting blood glucose levels of the transplanted mice were monitored weekly to assess diabetes reversal in vivo (Figure 1A); weight of the animals throughout the duration of the experiment can be found in Figure S2. All mice transplanted with NPIs showed a gradual decrease in hyperglycemia over the duration of the study. NPI graft recovery nephrectomies at 20‐weeks post‐transplant showed graft‐dependent function with all mice returning to a hyperglycemic state. The effect of host sex in NPI function and diabetes reversal was confirmed by measurement of IPGTT AUC, with females having a significantly lower AUC than males (Males: 361 ± 157, Females: 255 ± 99, *p = 0.0205*, Figure 1B). 94.4% of the female mice (17/18) transplanted with NPIs achieved normoglycemia compared to 73.6% of males (14/19) (*p = 0.0107*, Figure 1C), with a mean reversal time of 8 weeks for females and 12 weeks for males (*p = 0.0277*, Figure 1D).

In summary, diabetes reversal is achievable in both male and female mice upon NPI transplant under the kidney capsule; however, females reverse diabetes faster and at a higher rate than males, showcasing the effect of host sex on NPI maturation and function.

### 3.2 Neonatal Porcine Islets Have Improved Glucose Clearance in Females Compared to Males

**FIGURE 2:**
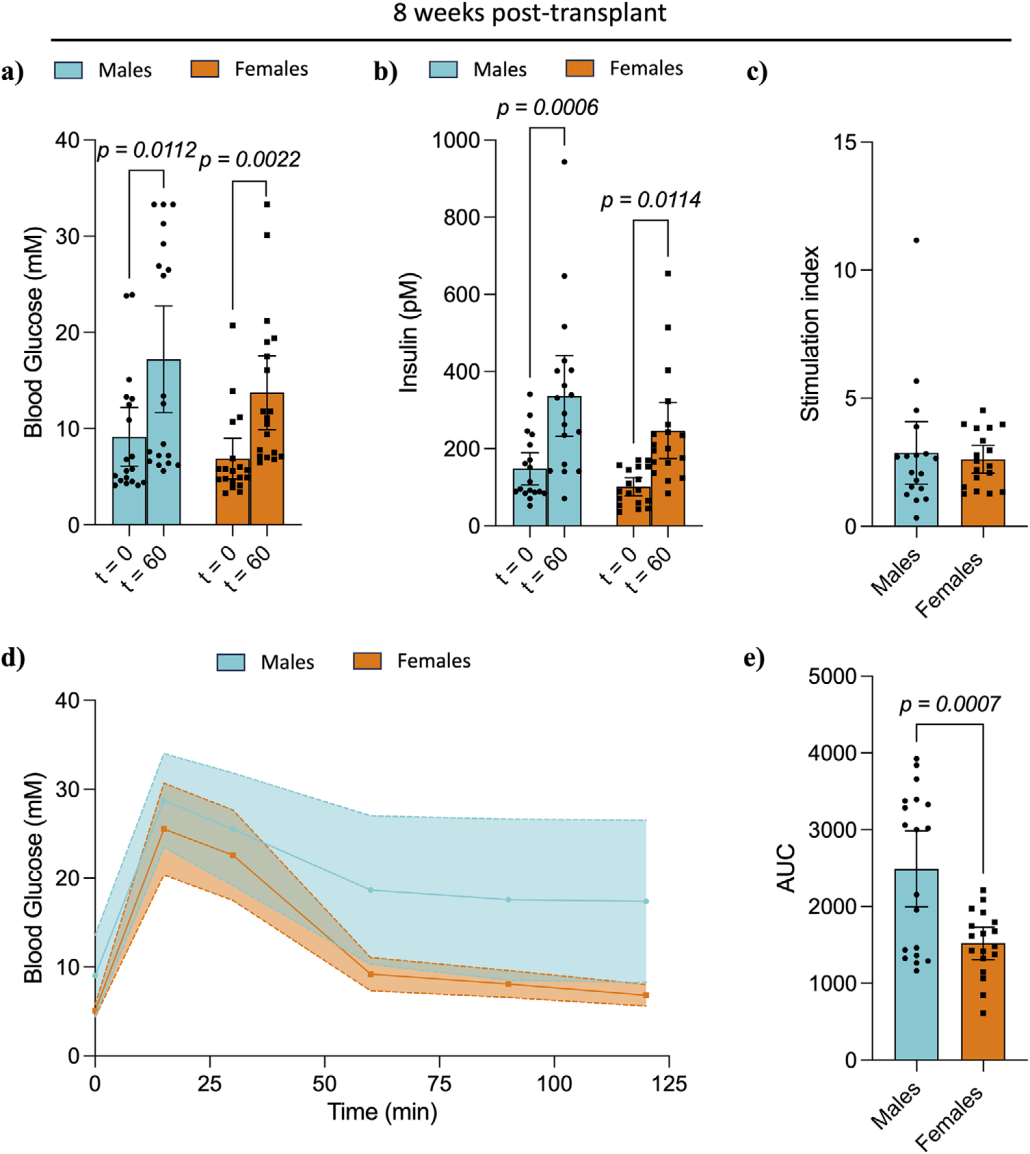
Intraperitoneal glucose challenge and stimulated porcine insulin secretion at 8 weeks post‐transplant. (a) Blood glucose levels, (b) porcine serum insulin levels, and (c) calculated stimulation index after an overnight fast and 60 min post‐intraperitoneal glucose administration (3 mg/g of weight). (d) Blood glucose levels during an IPGTT and (e) area under the curve for respective IPGTT. Statistical difference amongst groups was conducted using the unpaired *t*‐test with Welch's correction. Values are presented as means with standard deviation.

To understand the differences in NPI metabolic function, glucose challenges were performed at 8‐ and 20‐weeks post‐transplant to assess glucose clearance. IPGTTs performed at 8‐weeks post‐transplant showed that all animals displayed glucose‐responsive porcine insulin secretion, irrespective of sex. No statistically significant differences were found in the glucose or porcine insulin levels of fasting males and females prior to, or 60 min post‐glucose administration (Figure 2A,B). However, in all cases, there was a significant increase in blood glucose (Males: *p = 0.0112*, Females: *p = 0.0022*) and serum porcine insulin levels (Males: *p = 0.0006*, Females: *p = 0.0114*) 60 min post‐glucose administration compared to prior. No differences were found in the stimulation index between males and females (Figure 2C). Despite the similarities in glucose and serum porcine insulin levels between males and females, the IPGTT profiles were rather distinct, with both males and females showing an increase in blood glucose levels by 15 min, but with female mice demonstrating an accelerated glucose clearance, as indicated by the significantly lower area under the curve (AUC) measurements (Males: 2489 ± 1026, Females: 1521 ± 424, *p = 0.0007*, Figure 2D,E).

**FIGURE 3:**
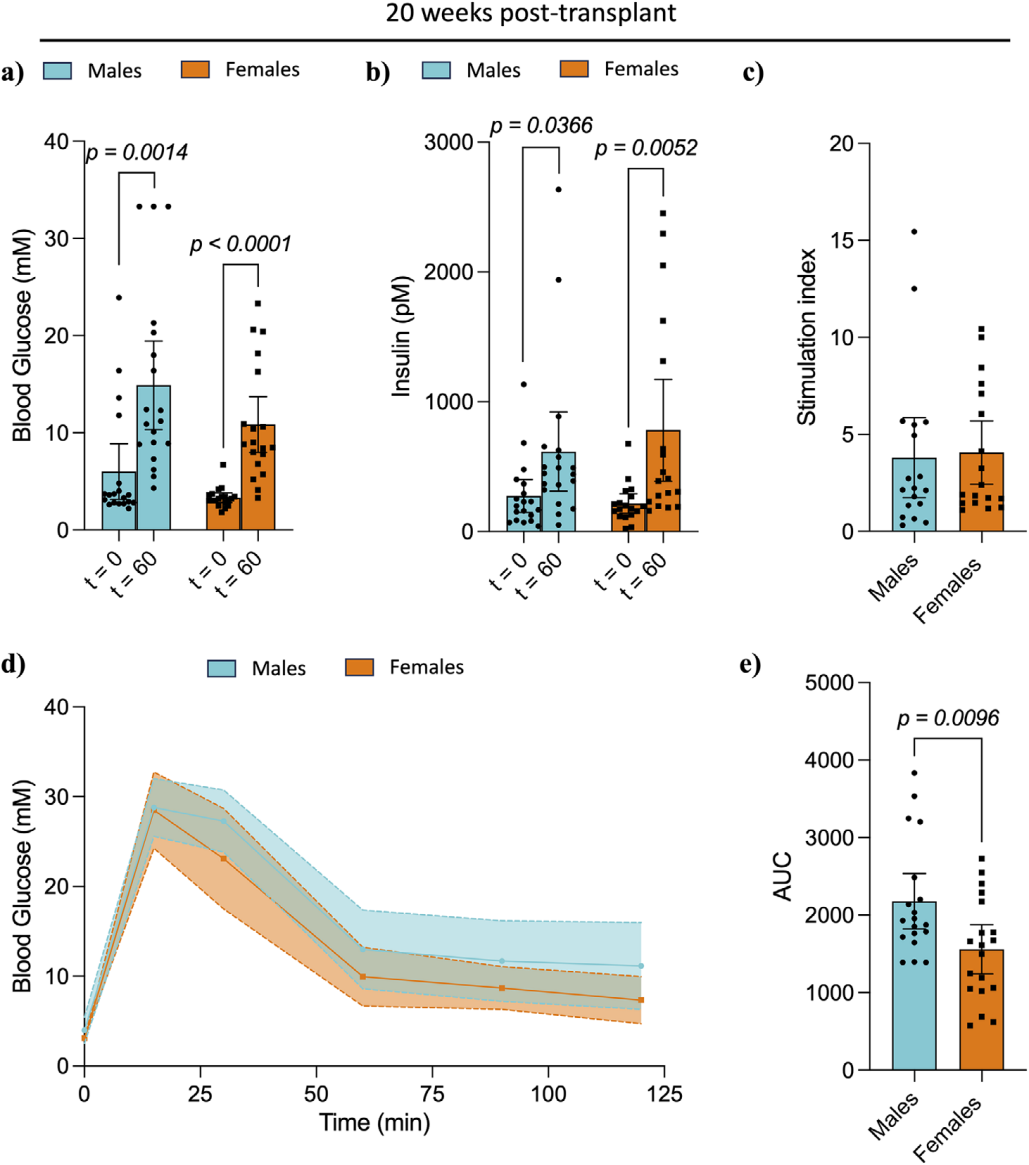
Intraperitoneal glucose challenge and stimulated porcine insulin secretion at 20 weeks post‐transplant. (a) Blood glucose levels, (b) porcine serum insulin levels, and (c) calculated stimulation index after an overnight fast and 60 min post‐intraperitoneal glucose administration (3 mg/g of weight). (d) Blood glucose levels during an IPGTT and (e) area under the curve for respective IPGTT. Statistical difference amongst groups was conducted using the unpaired *t*‐test with Welch's correction. Values are presented as means with standard deviation.

**FIGURE 4:**
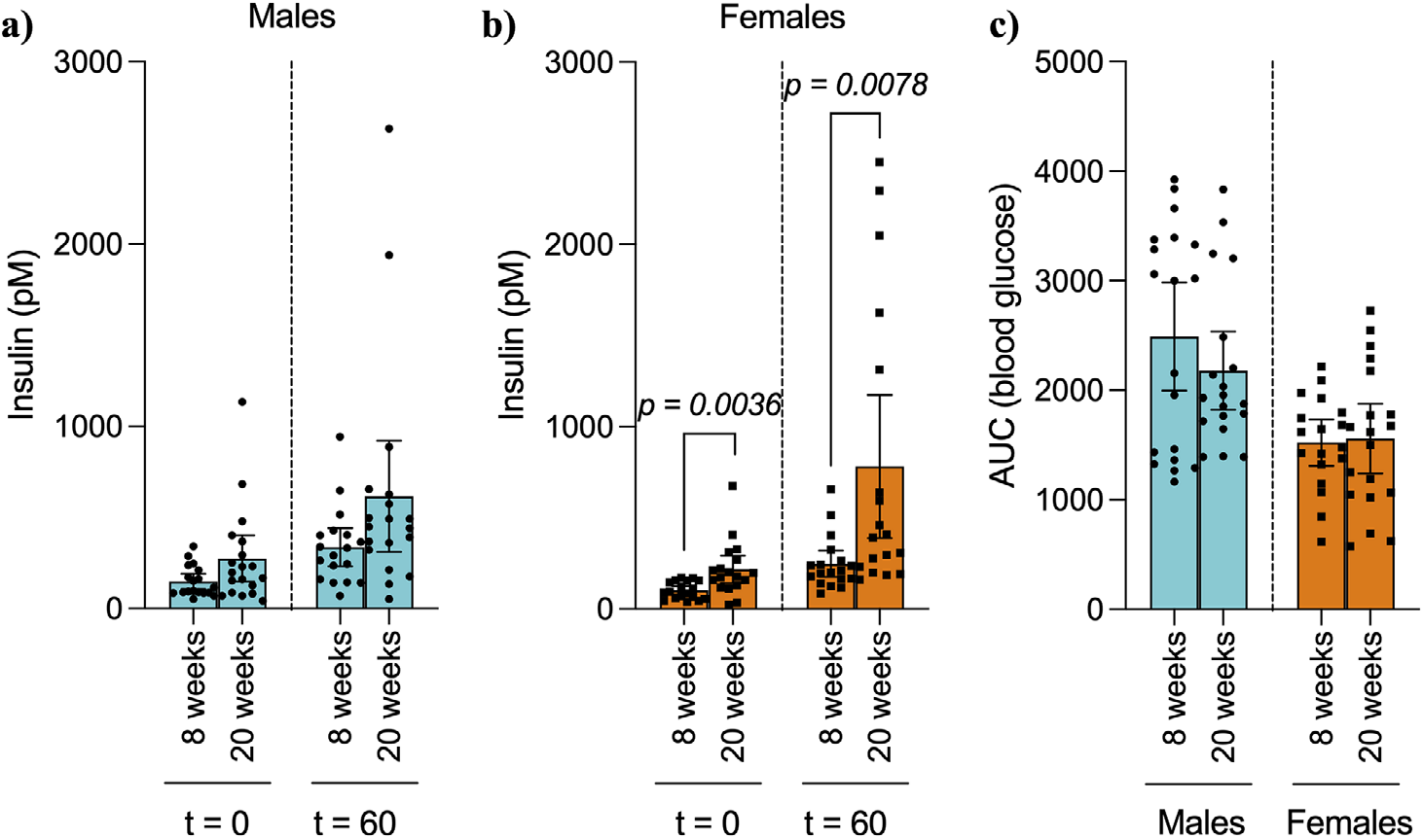
Comparison of basal and stimulated serum porcine insulin levels between 8‐ and 20‐weeks post‐transplant diabetic male and female B6.129S7‐Rag1^tm1Mom^ (B6/Rag^−/−^) mice transplanted with neonatal porcine islets under the kidney capsule. (a) Male and (b) female porcine insulin levels after an overnight fast and 60 min post‐glucose administration (3 mg/g of weight) via intraperitoneal injection at 8‐ and 20‐weeks post‐transplantation. (c) Determination of the significance was measured as the area under the curve of blood glucose levels after an overnight fast and 15, 30, 60, 90, and 120 min after glucose (3 mg/g of weight) administration at 8‐ and 20‐weeks post‐transplantation. Statistical difference amongst groups was conducted using the unpaired *t*‐test with Welch's correction.

IPGTT performed at 20‐weeks post‐transplant found no differences in blood glucose, porcine insulin levels, and stimulation index between males and females (Figure 3A‐C). However, similar to the 8‐week time‐point, female mice showed accelerated glucose clearance, as measured by AUC (Males: 2178 ± 740, Females: 1558 ± 655, *p = 0.0096*, Figure 3D,E). Interestingly, comparison of serum porcine insulin levels between 8‐ and 20‐weeks post‐transplant showed no significant differences in males, while female mice had significantly increased serum porcine insulin levels 20‐weeks post‐transplant in both fasting (8 weeks: 101 pM ± 47 pM, 20 weeks: 216 pM ± 149 pM, *p = 0.0036*) and 60 min post‐glucose administration (8 weeks: 246 pM ± 146 pM, 20 weeks: 781 pM ± 556 pM, *p = 0.0078*, Figure 4A,B). No differences were found in glucose clearance, measured as AUC, upon comparison of the different time points within each sex (Figure 4C).

### 3.3 Morphological Characterization and Insulin Content of Grafts

**FIGURE 5:**
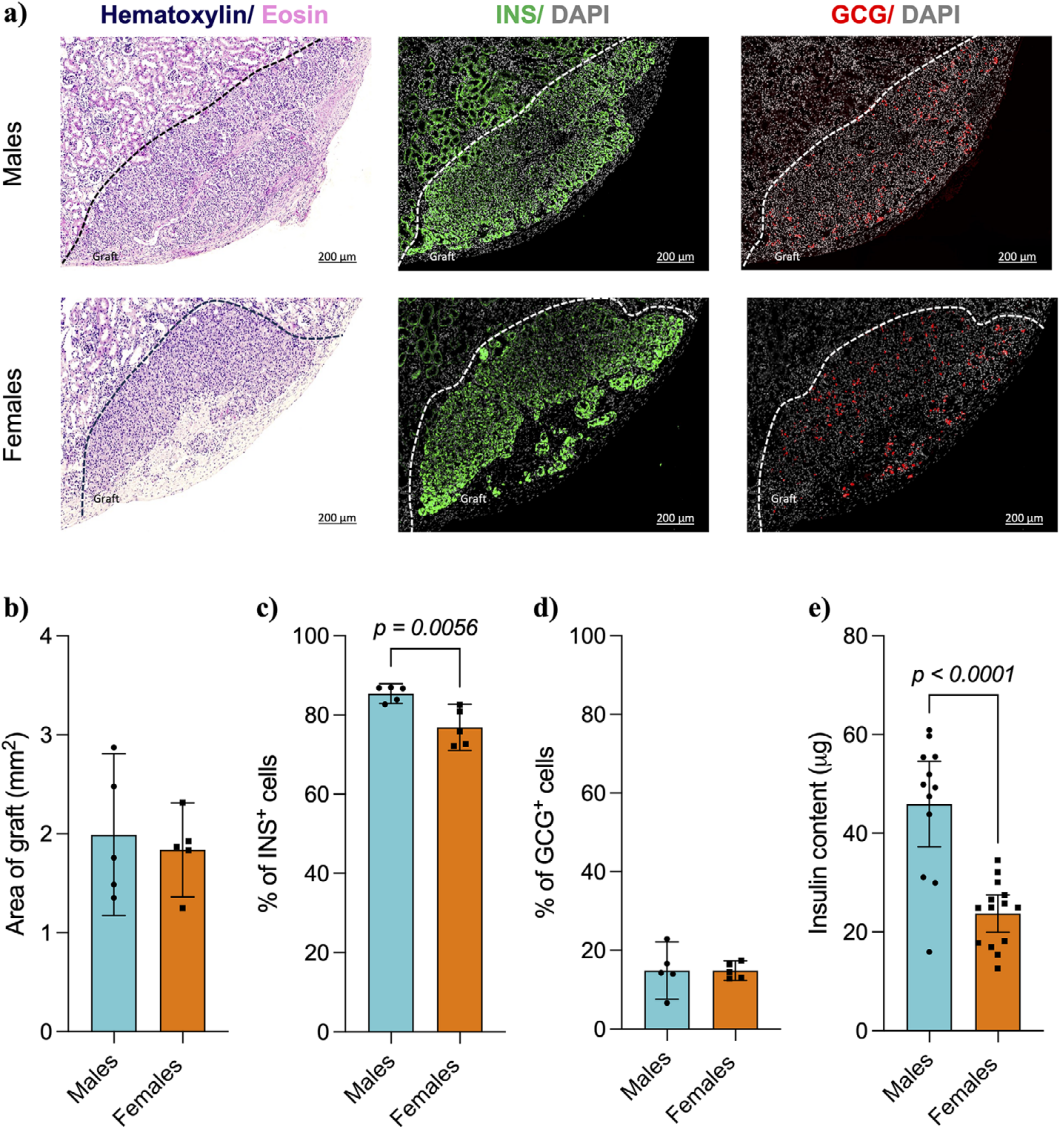
Morphological assessment of neonatal porcine islet grafts in diabetic male and female B6.129S7‐Rag1^tm1Mom^ (B6/Rag^−/−^) mice. (a) Representative images of hematoxylin/Eosin, insulin (INS), and glucagon (GCG) staining of the NPI grafts in male and female mice. (b) Grafts in male and female mice were quantified for total graft area (mm^2^), (c) % total insulin, and (d) glucagon‐positive cells. (e) Grafts in the male and female mice were also quantified for total cellular porcine insulin content (µg) Statistical difference amongst groups was conducted using the unpaired *t*‐test with Welch's correction. Values are presented as means with standard deviation.

Representative histological images of male and female grafts are illustrated in Figure 5A. Grafts were observed in all kidneys processed for histology (*n* = 5 per group). No statistically significant differences were found in the area of grafts measured between males and females (Figure 5B). Immunohistochemical assessment of the NPI grafts recovered from both male and female mice revealed insulin and glucagon‐positive cells. Insulin‐positive cells comprised the majority of the area in most grafts in both males and females; however, males had an increased percentage of insulin‐positive cells compared to females (Males: 85.3% ± 1.9%, Females: 76.8% ± 4.7%, *p = 0.0056*, Figure 5C). No statistically significant differences were observed in the percentage of glucagon‐positive cells between males and females (Figure 5D). Analysis of the insulin content of grafts (Males: *n* = 12, Females: *n* = 14) showed that grafts collected from male mice had significantly higher insulin content than those retrieved from female mice (Males: 45.9 µg ± 13.6, Females: 23.7 µg ± 6.5, *p < 0.0001*, Figure 5E).

## 4 Discussion

Latest research has shown the effect of sex on glucose homeostasis, the pathophysiology, incidence, and prevalence of diabetes, as well as the patient's response to treatment [17]. Similarly, islet and ß‐cell heterogeneity, as well as function under normal and stress conditions, differ between males and females [18]. Studying the relevance of host sex in ITx is vital for improving medical outcomes and addressing the ethical, legal, and social implications associated with this procedure. Ignoring these differences leads to inequalities in treatment and outcomes, which is why it is essential to consider sex as a key factor to ensure that advancements in ITx are equitable, safe, and effective for all patients. Herein, this study focuses on understanding the role of host sex in the function of NPIs transplanted under the kidney capsule of diabetic mice.

Robust glucose‐responsive porcine insulin secretion was observed at 8‐ and 20‐weeks after the transplant of NPIs under the kidney capsule of both male and female mice. Female mice had lower body weight throughout the experiment, which may contribute to lower insulin requirements and could partially explain the faster normalization of glucose homeostasis. However, our functional analyses indicate that NPIs in female recipients exhibit improved glucose clearance, which is an indicator of improved control of blood glucose levels, suggesting superior graft function. This improved glucose clearance correlated with an accelerated diabetes reversal as well as a diabetes reversal rate. Although insulin content was higher in male recipients, this did not translate into improved glucose responsiveness, suggesting potential differences in graft maturation or insulin release dynamics. These findings indicate that body weight alone does not fully account for the observed differences in diabetes reversal and that enhanced islet function in female recipients plays a key role. The activity of gonadal hormones (estrogens, androgens, and progesterones), especially after puberty, is one of the major causes of sex differences in physiology. The expression of Neurogenin‐3, a critical transcription factor for the differentiation and maturation of endocrine cells, is reduced upon the inhibition of estrogen receptor α, resulting in decreased ß‐cell proliferation in the developing mouse pancreas [19]. Similarly, glucose‐stimulated insulin secretion is increased upon the activation of estrogen receptor β in both humans and mice [20].

The immediate environment of the site of transplants has also been described to affect islet function. Female mice have previously been reported to have increased adipose tissue surrounding the kidney capsule [21], which might influence ß‐cell development and subsequent graft function. For example, adipsin, an adipokin synthesized in the adipose tissue and released into circulation, increases insulin secretion in response to glucose, preserves ß‐cells in diabetic mice, and is associated with protection from type 2 diabetes in humans [22, 23].

Despite the accelerated diabetes reversal and improved euglycemic rates in females, male mice exhibited a higher percentage of insulin‐positive cells as well as a higher porcine insulin graft content than females. Therefore, specific signals in the male host may influence the porcine insulin secretory response of NPIs. Through interactions with androgen and GLP‐1 receptors, testosterone increases glucose‐stimulated insulin secretion in both humans and mice [24]. These findings may also be attributed to variations in C‐peptide/ insulin clearance and insulin sensitivity between males and females, resulting in reduced secretory demand on the insulin‐producing cells in female grafts compared to those in male recipients. In fact, it is well‐established that both female humans and mice exhibit greater insulin sensitivity than males [25, 26].

Additional research will be necessary to elucidate the mechanisms responsible for faster glucose clearance and improved diabetes reversal following NPI transplantation in female mice compared to male mice. Further studies are also required to determine if our observations are applicable to other species and implantation sites. Nonetheless, our findings indicate that the function of NPIs is altered by the host sex, which should be considered in both preclinical rodent studies and ongoing clinical trials.

## 5 Conclusion

Overall, this study describes improved glucose clearance, accelerated diabetes reversal, and improved normoglycemic rates following the transplantation of NPIs in females compared to males. Despite reduced function, grafts recovered from male mice had a higher percentage of insulin‐producing cells as well as an increased insulin content, showcasing the relevance of understanding the role of sex as a biological variable to provide adequate, equitable medical care.

## Supporting information



Supporting information
