# Supplementary material for: Sex Differences in Maturation and Function of Neonatal Porcine Islets Upon Transplantation in Mice
Source: Xenotransplantation. 2025 Apr 17;32(2):e70039. doi: 10.1111/xen.70039 (PMC12005065; doi:10.1111/xen.70039)
Supplement: Supplementary file 1 — Supporting information [file XEN-32-e70039-s001.docx]

Supporting information for

**Sex Differences in Maturation and Function of Neonatal Porcine Islets upon Transplantation in Mice**

Cuesta-Gomez *et al.*

*Corresponding author. Email: [korbutt@ualberta.ca](mailto:korbutt@ualberta.ca)

**This PDF file includes:**

Figures S1 to S2

**Figure S1.** Morphological assessment of neonatal porcine islets. Representative image of isolated NPIs prior to transplant (A). Size of the isolated NPIs prior to transplant (B).

**
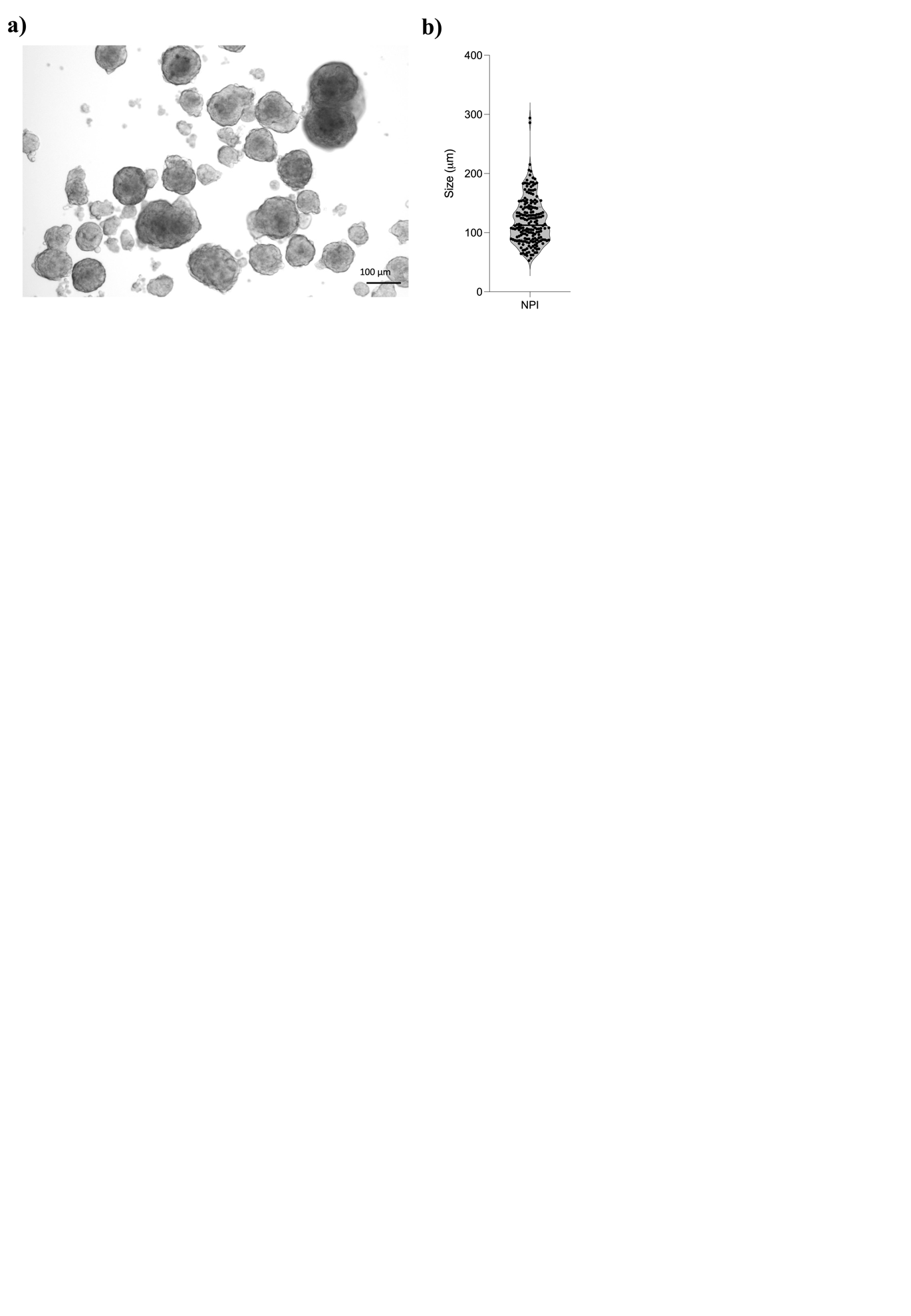
**

**Figure S2.** Health assessment through body weight monitoring. A) Body weight measurements throughout the experiment.

**
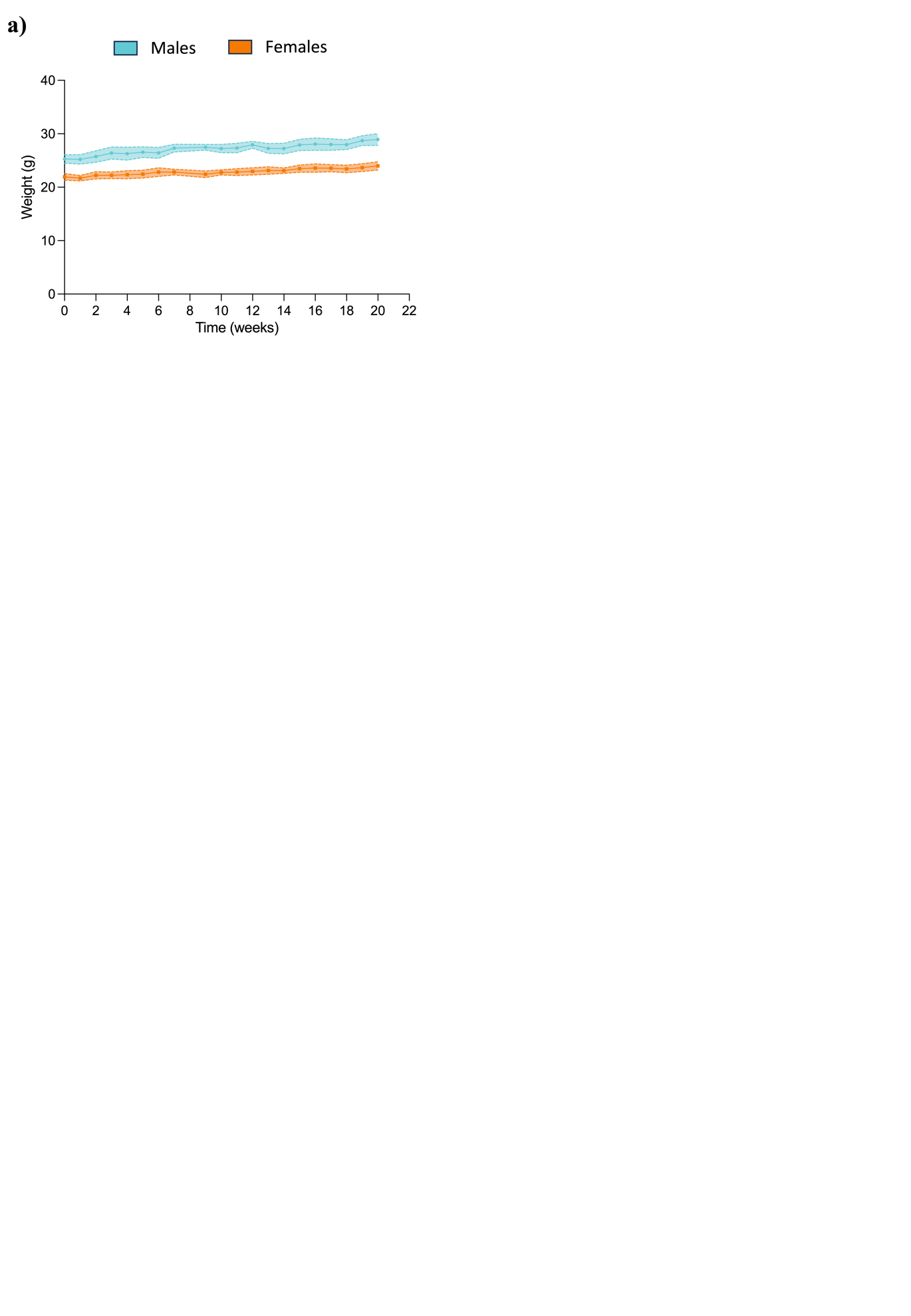
**
